# Response to the COVID-19 pandemic in the legally deprived population of liberty in Cali (Colombia): Miyerlandi Torres Agredo

**DOI:** 10.1093/eurpub/ckac131.082

**Published:** 2022-10-25

**Authors:** M Torres Agredo, J Tamayo, E Mina, L Luna, P Guerrero-Jaramillo, V Benavides-Cordoba

**Affiliations:** 1 Secretaría de Salud Publica Cali, Cali, Colombia; 2 Red de Salud del Centro, Empresa Social del Estado E.S.E., Cali, Colombia; 3 Centro Penitenciario Villahermosa, Cali, Colombia

## Abstract

With the COVID-19 pandemic, the challenge of reducing the transmission of the disease has led to new challenges in decision-making. The vulnerability of persons deprived of liberty led to the design of contagion mitigation alternatives. Since February 2020, at the Villahermosa Penitentiary and Prison Center in Cali, the Secretaría de Salud Pública (SSP) began a series of actions that intensified on March 11, when the WHO declared COVID -19 as a pandemic. The SSP, in an articulated work; configured a series of strategies aimed at mitigating the impact and speed of contagion, infectious disease doctors and internists were also summoned who provided recommendations and contributed to decision-making. An intervention model was designed, which was guided by two main processes: promotion and prevention actions and service provision actions. The articulated work and the high commitment of the actors involved, the development of strategies for biosecurity, hygiene, isolation, rapid detection, and, above all, immediate control of symptoms and medical care on-site with adjustments that allowed managing patients inside the institution; It has meant that to date mortality is below 1% and that for the time being the situation is under control.

**Key messages:**

• The humanization of health service provision is essential to achieve effective results.

• The rapid and articulated responses made it possible to maintain a mortality of less than 1% in this population.

